# Perceptions and Practices of Self-Medication among Medical Students in Coastal South India

**DOI:** 10.1371/journal.pone.0072247

**Published:** 2013-08-28

**Authors:** Nithin Kumar, Tanuj Kanchan, Bhaskaran Unnikrishnan, T. Rekha, Prasanna Mithra, Vaman Kulkarni, Mohan Kumar Papanna, Ramesh Holla, Surabhi Uppal

**Affiliations:** 1 Department of Community Medicine, Kasturba Medical College, Manipal University, Mangalore, Karnataka, India; 2 Department of Forensic Medicine, Kasturba Medical College, Manipal University, Mangalore, Karnataka, India; 3 Kasturba Medical College, Manipal University, Mangalore, Karnataka, India; Population Health and Preventive Medicine, Malaysia

## Abstract

Self-medication is a common practice worldwide and the irrational use of drugs is a cause of concern. This study assessed the prevalence of self-medication among the medical students in South India. The data was analysed using SPSS version 11.5. A total of 440 students were included in the study. The prevalence of self-medication was 78.6%. A larger number of females were self-medicating (81.2%) than males (75.3%). The majority of the students self-medicated because of the illness being too trivial for consultation (70.5%). Antipyretics were most commonly self–medicated by the participants (74.8%). Only 47% of the participants opined that self-medication was a part of self-care and it needs to be encouraged. 39.3% of the participants perceived that the supply of medicine without prescription by the pharmacist can prevent the growing trend of self-medication. Easy availability and accessibility to health care facilities remains the cornerstone for reducing the practice of self-medication.

## Introduction

According to William Osler, a great feature which distinguishes man from animals is the desire to take medicine [1]. Self-medication involves the use of medicinal products by the individuals to treat self-recognized disorders or symptoms, or the intermittent or continuous use of a medication prescribed by a physician for chronic or recurring diseases or symptoms [2]. Self-medication involves acquiring medicines without a prescription, resubmitting old prescriptions to purchase medicines, sharing medicines with relatives or members of one's social circle or using leftover medicines stored at home [3]. Self-medication thus forms an integral part of self-care, which can be defined as the primary public health resource in the health care system. It includes self-medication, non-drug self-treatment, social support in illness, and first aid in everyday life [2]. Not much is known about health-related problems and health care utilisation, including self-medication among young adults. The youth are highly influenced by the media and the internet which promote self-medication behaviour [4]. The increased advertising of pharmaceuticals poses a larger threat of self-medication to the younger population in general. This raises concerns of incorrect self-diagnosis, drug interaction, and use of drugs other than for the original indication [5]. The increase in the quantities and varieties of pharmaceuticals worldwide eases the accessibility of medicine by consumers, thereby giving options for its misuse. A study from Nigeria has observed self-medication as a common practice among group of health workers that included dental, midwifery and nursing students [6]. It has been suggested that self-prescription is also prevalent among practising physicians [7], [8]. A study conducted at All India Institute of Medical Sciences, New Delhi observed that self-medication was considerably high among undergraduate medical and paramedical students in India and it increased with medical knowledge [9]. There is a paucity of literature on the prevalence of self-medication among medical students and their attitude towards the same. The present study was hence, conducted to assess the prevalence of self-medication among the undergraduate students of Kasturba Medical College, Mangalore and to assess the students' perception and attitude regarding the practice of self-medication.

## Materials and Methods

Ethics Committee approval was obtained from the Institutional Ethics Committee of Kasturba Medical College, Mangalore (affiliated to Manipal University), India prior to the commencement of the study. This cross-sectional study was carried out among the undergraduate students of Kasturba Medical College, Mangalore during March–April, 2011. All the students of Manipal University are covered under a health insurance scheme, the benefits of which are restricted to free consultation and investigations in the hospitals associated with the University. The sample size was calculated assuming that 50% of medical students practice self-medication, and with 10% relative precision and 95% confidence interval the sample size was calculated to be 400. Accounting for a non-response error of 10%, final sample size was calculated to be 440. A pre designed semi structured questionnaire was used to collect the relevant information pertaining to the study variables. The questionnaire had 4 sections; Section A consisted of questions regarding age, gender, semester of the participating students and whether they self-medicated or not. If their response was in affirmative to self-medication they were instructed to fill Section B which consisted of questions regarding the practice of self-medication. Section C and Section D consisted of questions concerning their attitude and perception regarding self-medication. The students who did not self-medicate were instructed to fill in only Section C and D.The questionnaires were distributed to the medical undergraduates from the 1st, 2nd, 3rd and 4th year of MBBS after obtaining permission from the Dean of the Institution. The students were briefed on the aims and objectives of the study and a written informed consent was obtained from those who were willing to participate in the study. Authors, SU under the supervision of NK and PM who are faculty in the Department of Community Medicine at KMC, Mangalore administered the questionnaire during lecture hours to the participating students, chosen conveniently from respective MBBS batches. The questionnaires were assessed for their completeness and only the completed questionnaires were considered for the final analysis. The collected data was analysed using SPSS (Statistical Packages for Social Sciences) version 11.5. The results obtained were expressed in proportions.

## Results

A total of 440 students were assessed regarding their practice, attitude and perception regarding self –medication behaviour, out of which 43.2% (n = 190) were males and 56.8% (n = 250) were females. The mean age of the respondents was 20.3±1.5 years. The prevalence of self – medication among the study participants was 78.6% (n = 346). A proportionately larger number of females were self-medicating (n = 203, 81.2%) than males (n = 143, 75.3%).Self-medication was proportionately commoner in 3rd year of MBBS. The distribution of self-medication practice according to the year of MBBS is shown in Table 1.

**Table 1:** Characteristics of the study population (N = 440).

Variables	Self-medication	No self-medication	Total
	N = 346 (%)	N = 94 (%)	N (%)
Gender			
Male	143(75.3)	47(24.7)	190(100)
Female	203(81.2)	47(18.8)	250(100)
Year of MBBS			
1st year	78(71.6)	31(28.4)	109(100)
2nd year	98(78.4)	27(21.6)	125(100)
3rd year	103(87.3)	15(12.7)	118(100)
4th year	67(76.1)	21(23.9)	88(100)

Among the participants practicing self-medication, the majority (n = 320, 72.7%) followed allopathic system of medicine, followed by Homeopathic (n = 16, 3.6%) and Ayurvedic system of medicine (n = 10, 2.3%).

The majority of the students self-medicated because of the illness being too trivial for consultation (70.5%), followed by their confidence about the pharmacological knowledge (45%).More than half of the study participants (53.1%) used old prescriptions for the same illness as a source for information about the drug. The characteristics of the study subjects indulged in self-medication is shown in Table 2. Most of the participants (n = 322, 93%) checked the expiry date on the drugs before self-medication.

**Table 2:** Characteristics of study subjects indulged in self-medication (N = 346).

Variables	N (%)
Reasons for self-medication	
Illness too trivial for consultation	244(70.5)
Sufficient pharmacological knowledge	156(45.0)
To save time	66(19.0)
Avoid crowd at OPD	39(11.2)
Privacy	18(05.2)
Source of information about drugs	
Old prescription for same illness	184(53.1)
Academic knowledge	154(44.5)
Pharmacist	76(21.9)
Friends	64(18.5)
Drug advertisement/Internet	61(17.6)

Antipyretics were the most common class of drugs self –medicated by the majority of the participants (74.8%), followed by Antitussives (68.2%) and Analgesics (65.8%). It was also observed that 39.3% of the participants reported to have self-medicated themselves with Antibiotics [Table 3].

**Table 3:** Categories of drugs commonly self-prescribed (N = 346).

Categories	N (%)
Antipyretics	259(74.8)
Antitussives	236(68.2)
Analgesics	228(65.8)
Antihistamines	144(41.6)
Antibiotics	136(39.3)
Tonics/Vitamins	108(31.2)
Antidiarrhoel	106(30.6)
Antiemetics	75(21.6)
Antispasmodic	60(17.3)
Antiulcer	39(11.2)
Sedatives	29(08.3)

Beta lactam group (59.6%) was the most common class of antibiotics frequently self-medicated. Among the various indications for self-medication reported by the students [Table 4], fever was the most common (75.1%), followed by headache (64.7) and cough/cold (58.7). Sore throat (31.6%) was the most common indication for self-medication with antibiotics. 35.3% of the participants reported not to have completed the entire course of antibiotic regimen and stopped medication when the symptom subsided.

**Table 4:** Indications for self-medication (N = 346).

Indications	N (%)
Fever	260(75.1)
Headache	224(64.7)
Flu/Cough/Cold	203(58.7)
Pain	199(57.5)
Sore throat	139(40.1)
Vomiting	97(28.0)
Diarrhoea	92(26.6)
Ulcer in mouth	73(21.0)
Rash/allergies	70(20.2)
Insomnia	26(07.5)

Table 5 shows the attitude of the students towards the practice of self-medication. Only 47% of the participants opined that self-medication was a part of self-care and it needs to be encouraged. 39.3% of the participants perceived that the supply of medicine without prescription by the pharmacist can prevent the growing trend of self-medication [Table 6].

**Table 5:** Attitude of the students towards self-medication (N = 440).

Items	Yes	No	Not sure
	N (%)	N (%)	N (%)
Self-medication is a part of self-care	207(47.0)	224(50.9)	09(02.0)
Continue with/start self-medication	250(56.8)	140(31.8)	50(11.4)
Advice self-medication to friends	140(31.8)	300(68.2)	00(00.0)

**Table 6:** Perception of students regarding methods to prevent the growing trend of self-medication (N = 440).

Items	N (%)
Prevent the supply of medicines without prescription	173(39.3)
Awareness and education regarding implications of self-medication	123(28.0)
Enforcing strict rules regarding misleading pharmaceutical advertising	91(20.7)
Working towards making health care facilities easily available	35(08.0)
No opinion	18(04.1)

## Discussion

The International Pharmaceutical Federation defines self-medication as the use of non-prescription medicines by people on their own initiative [10]. As per the World Health Organization [11], “Self-medication is the selection and use of medicines by individuals to treat self-recognised illnesses or symptoms.” Self-medication is considered an element of self-care [11]. Self-care, including self-medication, has been a feature of healthcare for many years and people have always been keen to accept more personal responsibility for their health status [10]. Self-medication by itself has both pros and cons that depend on who and what one choses to self-medicate [12]. The present study was conducted to evaluate the practices, attitude and perception of self-medication among medical students. The prevalence of self-medication in our study was found to be 78.6%. In studies conducted within India, the prevalence of self-medication among the medical students was shown to be ranging between 57.1% and 92% [13]–[15]. Other studies on Indian students from non-medical background showed a prevalence of 80.1% in Tamil Nadu [16] and 87% in Uttar Pradesh [17]. In studies conducted in developing countries, the prevalence of self-medication was shown to be 25.4% and 43.2% in Ethiopia [18], [19], 51% in Slovenia [20], 55.3% in Pakistan [21], 55% in Egypt [22], 56.9% in Nigeria [23] and 80.9% in Malaysia [24]. Similarly, a nine-year follow-up study of a nationwide sample from Norway has reported a self-prescribing behaviour among young doctors [8]. Gender is considered as an important factor in self-medication patterns among young adults including students. The prevalence of self-medication was observed to be higher among females in our study. Similar observations were made in studies from India [13], [15] and abroad [20]. The majority of the study participants followed allopathic system of medicine which is similar to the observations made in other studies from India [17], [25].

In our study the most common reason for self-medication reported by a large number of participants was the illness being too trivial. Similar observations were reported in a few studies from India [13], [15]. However, in a study from Tamil Nadu [16] most students practiced self-medication as it was time saving, whereas in Punjab [25] the most common reason for self-medication was for quick relief. Students are prone to make unsupervised health-related decisions especially students of health sciences who feel confident of their knowledge about the drugs. In the present study, 45% of the participants indulged in self-medication owing to their sufficient pharmacological knowledge. In studies from Ethiopia [18], [19] Karachi [21], and Malaysia [24] prior experience with the illness was observed to be the most common reason for self-medication. Previous prescription for the same illness was reported as the most common source of information about the drugs used for self-medication in the present study, which was similar to observation made in Tamil Nadu [16] and Uttar Pradesh [17]. However, in another study from India [15], and Ethiopia [18] textbooks were reported as the most common source of information.

Antipyretics were the most common class of drugs self-medicated by majority of the participants in our study. Similar observations were made in a study from South India [15] and Ethiopia [18]. However, in studies from Iran [26], Mozambique [27], Pakistan [21], and Egypt [22] analgesics were the most common group of drugs self-medicated. Fever was the most common indication for self-medication in our study which was similar to observations made in Tamil Nadu [16]. However, in studies from Western [13] and Southern part of India [15], cough & cold was the most common symptom for self-medication. A study from Ethiopia [18] reported fever as the most common symptom for self-medication.

Antibiotics were self-medicated by 39.3% of the study participants in our study. Our results are higher than that reported in other studies from India [13], [14]. The antibiotic use for self-medication was reportedly similar, and higher in studies from developing countries [22], [23], [28]. Beta lactams were the most common class of antibiotics frequently self-medicated in our study. Similar observations were reported in studies conducted exclusively on self-medication with antibiotics [23], [28]–[30].Beta-lactams were most commonly used for self-medication. Sore throat was the most common indication for antibiotic use in our study. Similar observation was reported in a study from China [31] and Europe [32]. In studies from Nigeria [23], [28], diarrhoea and gastro-intestinal infections were reported as the most common indication for antibiotic use whereas in Turkey [33] and Greece [30], common cold was the most common indication for antibiotic use.

In the present study 47% of the participants felt that self-medication was part of self-care which was higher to that reported in studies from Ethiopia [19] and Karachi [21]. More than50% of the participants wished to continue with self-medication/start self-medication. Nearly one-third of them were even ready to advice self-medication to their friends. The irrational use of drugs is a cause of public and professional concern [3]. Self-medication as part of self- care can be justified only when there is a judicial use of medicines. There is always a risk of using expired drugs, sharing them with friends or taking medicine that have been originally prescribed for some other problem. Irrational use of drugs may result in accidental drug poisoning. Other problems related to self-medication are wastage of resources and serious health hazards such as drug dependence, adverse reaction and prolonged suffering. Antimicrobial resistance is another problem worldwide particularly in developing countries where antibiotics are often available without a prescription [34]. Lack of general knowledge on correct antibiotic use has been observed among students in a study from Portugal [35]. In a telephone based population survey in the USA, it was observed that 58% of the participants were not aware of the possible health danger associated with antibiotic use [36].

The present study perceives that to prevent the growing trend of self-medication, strong policies should be applied prohibiting the supply of medicines without a valid prescription. The youth, especially the females should be educated and made aware about the implications of self-medication. The study findings are based on a single centre study in coastal South India and hence, the study observations cannot be generalized per se. More multi-centric studies need to be carried out among medical students and general population at large to understand the various factors influencing the practice of self-medication in India. The role of socio-economic status and its influence on practice of self-medication needs to be explored in future studies.
